# Novel Hypomorphic Alleles of the Mouse Tyrosinase Gene Induced by CRISPR-Cas9 Nucleases Cause Non-Albino Pigmentation Phenotypes

**DOI:** 10.1371/journal.pone.0155812

**Published:** 2016-05-25

**Authors:** Anil K. Challa, Evan R. Boitet, Ashley N. Turner, Larry W. Johnson, Daniel Kennedy, Ethan R. Downs, Katherine M. Hymel, Alecia K. Gross, Robert A. Kesterson

**Affiliations:** 1 Department of Genetics, University of Alabama at Birmingham, Birmingham, Alabama, United States of America; 2 Department of Optometry and Vision Sciences, University of Alabama at Birmingham, Birmingham, Alabama, United States of America; 3 Science and Technology Honors Program, University of Alabama at Birmingham, Birmingham, Alabama, United States of America; Montana State University, UNITED STATES

## Abstract

Tyrosinase is a key enzyme in melanin biosynthesis. Mutations in the gene encoding tyrosinase (*Tyr*) cause oculocutaneous albinism (OCA1) in humans. Alleles of the *Tyr* gene have been useful in studying pigment biology and coat color formation. Over 100 different *Tyr* alleles have been reported in mice, of which ≈24% are spontaneous mutations, ≈60% are radiation-induced, and the remaining alleles were obtained by chemical mutagenesis and gene targeting. Therefore, most mutations were random and could not be predicted *a priori*. Using the CRISPR-Cas9 system, we targeted two distinct regions of exon 1 to induce pigmentation changes and used an *in vivo* visual phenotype along with heteroduplex mobility assays (HMA) as readouts of CRISPR-Cas9 activity. Most of the mutant alleles result in complete loss of tyrosinase activity leading to an albino phenotype. In this study, we describe two novel in-frame deletion alleles of *Tyr*, *dhoosara* (Sanskrit for gray) and *chandana* (Sanskrit for sandalwood). These alleles are hypomorphic and show lighter pigmentation phenotypes of the body and eyes. This study demonstrates the utility of CRISPR-Cas9 system in generating domain-specific in-frame deletions and helps gain further insights into structure-function of *Tyr* gene.

## Introduction

The *Tyr* gene functions in the melanin synthesis pathway. There are over 250 mutations in the *Tyr* gene that cause oculocutaneous albinism (OCA1) in humans (http://www.ifpcs.org/albinism/oca1mut.html)). Similar phenotypes occur in mice with disruptions in the *Tyr* gene. There are over 100 alleles of *Tyr* listed in the Mouse Genome Informatics resource (http://www.informatics.jax.org/marker/MGI:98880). Most are radiation-induced mutations (60%), spontaneous mutations (24%), or chemically induced mutations (~14%). Exact sequence information of the mutant alleles is known only for 21 of these MGI alleles since all of the above alleles were generated and identified using random mutagenesis and phenotypic screening. Custom-designed nucleases are useful in generating mutations in targeted regions of the gene and genome. With the advent of the CRISPR-Cas9 system, a number of targeted alleles are being rapidly generated, not only in the coding region of the gene [1, 2] but also in the non-coding regulatory regions [3]. While most mutations are null alleles causing a complete loss of pigmentation (albinism), there are mutations that are hypomorphic alleles resulting in lesser pigmentation (S1 Table).

In order to identify additional alleles of the mouse *Tyr* gene for use as reference alleles in targeted gene disruption, we used the CRISPR-Cas9 nuclease system to target two specific sites of exon 1. The first site is close to a well-known albino mutation, Tyr^*c-2J*^ (R77L) (Le Fur, Kelsall, Mintz, 1996). The albino/R77L allele is a spontaneous mutation resulting in an albino coat color, which is attributed to a complete loss of enzyme activity in melanosomes and the retinal pigment epithelium (RPE) [4]. Tyrosinase Arginine 77 is highly conserved in vertebrates (and in tyrosinase-related proteins) wherein the highly charged residue is closer to the N-terminus in a region forming a short helix. This region is distant and distinct from the catalytic sites. Substituting the charged Arginine77 with the hydrophobic Leucine (R77L) is likely to result in the loss of allosteric interactions, thereby abrogating tyrosinase enzyme function. The important role of Arginine residues in enzyme function and protein structure has been well documented in literature [5, 6].

The second site we targeted is in close proximity to the catalytic site (aa 202–219; HEAPGFLPWHRLFLLLWE; Prosite PS00497) at the N-terminal of the enzyme. Tyrosinase binds two copper ions (CuA and CuB), and the signature pattern located in the CuA-binding region at the N-terminus of the enzyme contains two histidine residues. Since mutations in either of these regions is likely to affect enzyme function, we sought to study the consequences of creating mutations in the first of these two copper-binding regions encoded in exon 1 using the CRISPR-Cas9 system.

## Materials and Methods

### CRISPR/sgRNA design and synthesis, and Cas9 mRNA synthesis

CRISPR targets were identified in exon 1 of the mouse *Tyr* gene (ENSMUSG00000004651) using an online design tool ([7]; crispr.mit.edu). Guide sequences with high scores that indicated low number of off-target sites were chosen at the two regions. A region proximal to the 5’ end of exon 1 that includes a common albino mutation (Tyr*c-2j;* G291T > R77L) was targeted by one guide sequence (5’ CRISPR), ACGGTCATCCACCCCTTTGA (*AGG*), while a downstream region that is highly conserved in many polyphenol oxidases/tyrosinases was targeted by a second guide sequence (3’ CRISPR), AAGAAATTCGAGAACTAACT (*GGG*). Guide RNA molecules were generated by cloning annealed oligonucleotides into the DR274 vector as described by Hwang and co-workers [8]. Positive clones were verified by Sanger sequencing, linearized with HindIII enzyme and used as templates for *in vitro* transcription reactions using the T7 Ampliscribe Kit (Epicenter, Madison WI).

Cas9 mRNA was *in vitro* transcribed using NotI linearized pCS2-nCas9n plasmid as template [9]. *In vitro* transcription, capping and polyA tailing reactions were performed using the SP6 mScript *in vitro* transcription kit (CellScript, Madison WI).

### Mouse breeding, microinjection into zygotes and blastocyst culture

All experiments were performed in accordance with the recommendations in the *Guide for the Care and Use of Laboratory Animals* published by the National Institutes of Health. The protocols used were approved and conducted according to the University of Alabama at Birmingham IACUC. We bred “black” C57BL/6J male mice with “albino” Tyr^-^ C57BL/6J (homozygous for Tyr^*c-2j*^ allele) female mice to produce heterozygous zygotes, which would have a black coat color without any further modification. Pronuclear injections into these heterozygous zygotes were performed with a solution of both sgRNAs (50 ng/μl each) and Cas9 mRNA (100 ng/μl). Injected zygotes were cultured for 3 days in KSOM medium to the blastocyst stage, or implanted into pseudopregnant CD1 recipients. Genomic DNA obtained from cultured blastocysts or tail biopsies of putative founder animals were assessed for the presence of mutations in the tyrosinase gene. Animals harboring mutant alleles were bred to Tyr^-^ C57BL/6J albino (Tyr^*c-2j*^) mice to obtain F1 animals and assess germline transmission.

### Detecting the presence of indels

Genomic DNA from single cultured blastocysts was used for polymerase chain reactions (PCRs). PCR products were analyzed using a heteroduplex mobility assay or HMA [10, 11] for assessing the nuclease activity (“blastocyst assay”). Briefly, to obtain genomic DNA from cultured blastocysts (~3 days after injection), single blastocysts were placed in 10μl of lysis solution (containing proteinase K) and incubated at 55°C for 2 hours, followed by 95°C for 10 minutes to inactivate the proteinase K. A small aliquot (0.5–1 μl) of this solution was directly used as a PCR template to amplify a region flanking the CRISPR target site.

Genomic DNA from mouse-tail biopsies was obtained by digesting in lysis buffer (50 mM Tris-HCl pH 8.0, 100 mM EDTA pH 8.0, 100 mM NaCl, 1% SDS) with proteinase K (0.3 mg/ml).

PCRs were set up using the following oligonucleotide primers: for 5’CRISPR, 5’Tyr_F: 5’-CTCTGATGGCCATTTTCCTC-3’ and 5’Tyr_-R 5’-AACCCATGAAGTTGCCTGAG-3’ to obtain a 248 bp fragment; for 3’CRISPR, 3’Tyr_F 5’-ATGAAGCACCAGGGTTTCTG-3’ and 3’Tyr_R 5’-AAGGATGCTGGGCTGAGTAAG-3’ to obtain a 199 bp fragment. The amplicons were subjected to denaturation-slow renaturation to facilitate formation of heteroduplexes using a thermocycler. These samples were then resolved on polyacrylamide gels (6%) and the resulting mobility profiles used to infer efficiency of CRISPR-Cas9 nuclease activity.

Once indels were detected by HMA from tail genomic DNA of potential founder animals, PCR using outer primers (5’Tyr_F and 3’Tyr_R) was performed to amplify fragments (759 bp) that included both CRISPR target regions. The amplified products were cloned using the TOPO-TA cloning kit (Invitrogen, Carlsbad CA). Ten representative colonies were picked from each plate and grown in 1.5 ml liquid cultures to isolate plasmid DNA. Plasmid DNA was sequenced using M13 F and R primers.

### Sample preparation and imaging of retinal sections and RPE flat mounts

Eyeballs enucleated from 2 month old mice of either sex were placed in 4% paraformaldehyde (PAF) in 0.1 M PBS, pH 7.4 (PBS) for 2 hours at room temperature. Following PAF treatment, one eyeball from a single animal was incubated overnight in 30% sucrose in PBS at 4°C and then embedded in Tissue-Tek O.C.T. (Sakura Finetek USA), frozen, and sectioned at 12 micron intervals using a cryomicrotome at 19°C. Cryosections were mounted in 90% glycerol in PBS. A retinal pigment epithelium (RPE) wholemount was prepared using the contralateral eyeball as previously described ([12]).

Images of cryosectioned eyeballs were collected in brightfield on a Nikon Eclipse TE2000S at 20X magnification. RPE wholemount images were collected on a Nikon SMZ800 dissecting microscope. All images captured using a Qimaging micropublisher 3.3 with real time viewing camera.

### Quantification of pigmentation and statistical analysis

Pigment levels between animal groups were determined using RPE wholemounts imaged on a Nikon Eclipse TE2000S at 10X magnification. Brightfield images captured were converted to grayscale and background was subtracted. After inverting colors, a mean gray value was determined using six measurements from 50x50 micron regions of interest. All image manipulation was conducted using ImageJ (Fiji). Data are presented in bar chart as mean and standard error of the mean. Statistical comparisons for parametric continuous data were made using student’s t-test (p<0.05). All statistical analyses were performed using JMP 12 software (SAS institute).

### Analysis of protein expression by Western Blotting

Tissue samples of adult skin were homogenized by sonication in NP-40 lysis buffer (1% NP-40, 150mM sodium chloride, 50mM Tris-HCl pH 8.0, 2mM EDTA, 1mg/mL aprotinin, 1mM PMSF). Protein concentrations were determined using Pierce BCA Protein Assay Kit (Thermo Scientific, Rockford, IL). Proteins (20 μg total per lane) were separated by reducing sodium dodecyl sulfate-polyacrylamide gel electrophoresis and transferred to PVDF membrane overnight. The membranes were then blocked in 5% bovine serum albumin for 2 hours at room temperature and probed with the antibody against tyrosinase (1:2,000, ab180753, Abcam, Cambridge, UK), followed by incubation with a horseradish peroxidase-coupled anti-rabbit IgG antibody (1:5,000, ab97051, Abcam, Cambridge, UK). Protein bands were visualized via Pierce ECL 2 Western Blotting substrate (#80196, Thermo Scientific, Rockford, IL). Equal loading was verified using an antibody against GAPDH (1:10,000, 5174, Cell Signaling Technology, Danvers, MA). Equal loading was verified using an antibody against alpha-tubulin (1:20,000, ab4074, Abcam, Cambridge, UK).

## Results

### Testing and validating CRISPR/sgRNA nuclease activity

**Fig 1:**
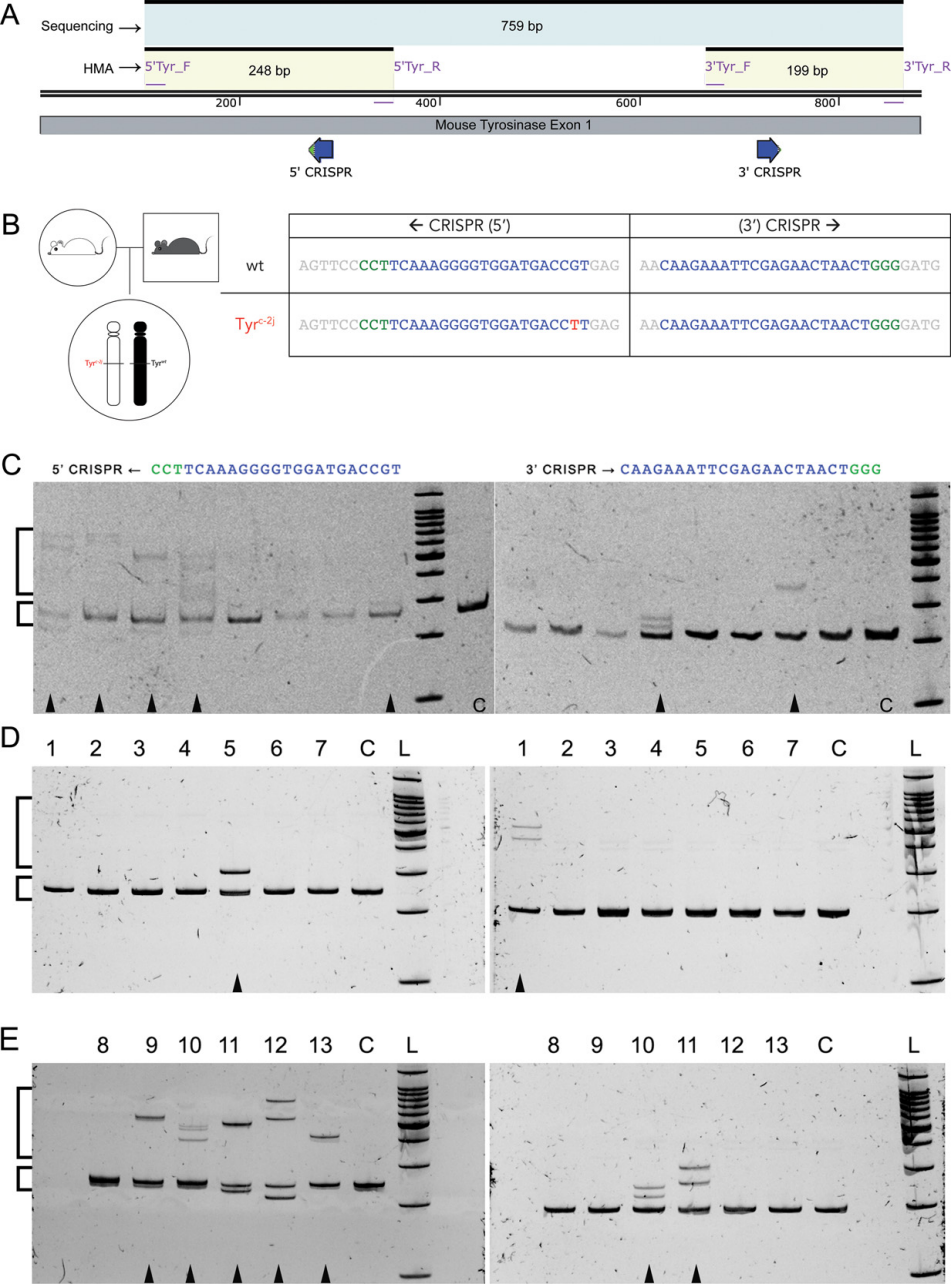
CRISPR targeting and mutation detection by heteroduplex mobility assay in the *Tyr* gene. (A) Schematic showing CRISPR targeting regions (blue bold arrows), PCR primer binding sites, and amplicon sizes. (B) Breeding scheme with genotype of the zygotes used for CRISPR-Cas9 injections, and the CRISPR target sequences on both chromosomes. (C, D, E) Images of ethidium bromide stained polyacrylamide gels (6%) showing separation of homoduplex and heteroduplex PCR amplicons from CRISPR-Cas9 RNA injected, single cultured blastocysts (C, arrowheads) and tail DNA of potential founder mice (D, E). Gels on the left correspond to the 5’CRISPR target site, and those on the right correspond to the 3’CRISPR target site. Small and large square brackets indicate homoduplex and heteroduplex bands, respectively. L = 100 bp ladder; C = uninjected wildtype control.

We employed a two-step workflow to validate CRISPR/sgRNAs and raise potential founders. Two distinct CRISPR/sgRNAs were synthesized and co-injected with Cas9 mRNA to target 5’ and 3’ regions of mouse *Tyr* exon 1 (Fig 1A and 1B). Heterozygous zygotes (Tyr^+/c-2j^, black) obtained from breeding C57BL/6 (Tyr^+/+^black) mice with albino C57BL/6J (Tyr^c-2j/c-2j^) mice were injected with the CRISPR/sgRNA-Cas9 RNA mixture (Fig 1B). This scheme was used to ensure that a single hit by either of the CRISPR/sgRNAs in the wildtype allele would result in mutations that potentially give an easily scorable pigmentation phenotype.

HMA profiles of two independent *Tyr* PCR amplicons from genomic DNA of injected zygotes cultures into blastocysts showed that the 5’ CRISPR/sgRNA caused double strand breaks, resulting in indels due to non-homologous end joining (NHEJ) in 5/8 cases (62.5%), while the 3’ CRISPR/sgRNA showed nuclease activity in 2/8 (25%) cases (Fig 1C). These results validated the nuclease activity of the two CRISPR/sgRNAs to help create mutations in F_0_ animals.

### Generation of founder (F_0_) animals with mutations induced by validated CRISPR/sgRNA

Pronuclear injections with both the CRISPR/sgRNAs followed by transfer of 32 zygotes into two CD1 pseudo-pregnant recipient mice were performed to obtain potential founder animals with mutations in the *Tyr* gene. We obtained 13 pups, which fell into four groups based on pigmentation: 7 (54%) black, 3 (23%) mosaic (black and albino), 1 (8%) non-albino, and 2 (15%) albino (Tables 1 and 2). The non-albino mouse had mild pigmentation, which could be easily distinguished from the albino mice.

**Table 1:** Details of the F0 animals obtained from CRISPR-Cas9 injections.

#	Sex	Coat color	HMA profile;Indels (“+” = insertion; “-” = deletion)		F1
			5’ CRISPR	3’ CRISPR	
1	F	Black	None	HMA;-5 bp	NA
2	M	Black	None	None	NA
3	M	Black	None	None	NA
4	M	Black	None	None	NA
5	M	Black	HMA;+1 bp, -4 bp (wt)	None	NA
6	F	Black	None	None	NA
7	M	Black	None	None	NA
8	M	Mosaic	None	None	None
9	M	Mosaic	HMA;-10 bp (wt)	None	2 black + 3 albino
10	M	Mosaic	HMA; +5 bp, -13 bp (wt)	HMA; -2 bp (wt)	3 black + 2 albino
11	M	Non-albino	HMA; -15 bp,-2 bp (c-2j)	HMA; -3 bp (wt),-3 bp (c-2j)	3 albino + 4 non-albino
12	F	Albino	HMA; -29 bp (wt),+1 bp, -3 bp (c-2j)	None	8 albino
13	F	Albino	HMA; +178 bp, -23 bp	None	6 albino

Sex (F = female, M = male), coat color (black, mosaic, non-albino and albino), presence or absence of positive HMA profiles (with heteroduplxes indicating indels), nature of indels (+ = insertion,— = deletion) obtained from sequencing cloned PCR products, and germline transmission of Tyr mutant alleles. (wt) and (c-2j) refer to the wildtype or mutant allele/chromosome in the background. NA = not applicable (not bred).

**Table 2:** Multiples mutant alleles resulting from NHEJ at 5’ and 3’ CRISPR target sites.

	← 5’ CRISPR	Indels	Protein	Phenotype
wt	GGACCTCAGTTCC*CCT*T CAAAGGGGTGGATGACCGTGAGTCCTGG			
	-G--P--Q--F--P--F- -K--G--V--D--D--R--E--S--W-			
Tyr^c-2j^	GGACCTCAGTTCC*CCT*T CAAAGGGGTGGATGACC**T**TGAGTCCTGG			
	-G--P--Q--F--P--F- -K--G--V--D--D--L--E--S--W-			
5 (wt)	GGACCTCAGTTCCCCT**C** ----AAGGGGTGGATGACCGTGAGTCCTGG	+1, -4	In frame deletion	Black
	-G--P--Q--F--P--S- -G--V--D--D--R--E--S--W-	ΔFK-->S		
9 (wt)	GGACCTCAGTTCCCCT ----------GGATGACCGTGAGTCCTGG	-10	Premature stop	Mosaic
	-G--P--Q--F--P--W--M--T--V--S--P-	44 aa* + stop		
10 (wt)	GGACCTCA**TCCAC**-------------GGGGTGGATGACCGTGAGTCCTGG	+5, -13	Premature stop	Mosaic
	-G--P--H--P--R--G--G--*--P--*--V--L-	4 aa* + stop		
11 (wt)	GGACCTCAGTT ---------------GGATGACCGTGAGTCCTGG	-15	In frame deletion	Non-albino
	-G--P--Q--L- -D--D--R--E--S--W-	5 aa deletion		
11 (Tyr^c-2j^)	GGACCTCAGTTCCCCTT--AAGGGGTGGATGACCTTGAGTCCTGG	-2	Premature stop	
	-G--P--Q--F--P--L- -R--G--G--*--P--*--V--L-	3 aa* + stop		
12 (wt)	GGACC -----------------------------GTGAGTCCTGG	-29	Premature stop	Albino
	-G--P- -*--V--L-	Stop		
12 (Tyr^c-2j^)	GGACCTCAGTTCCCCT**C** ---AAGGGGTGGATGACCTTGAGTCCTGG	+1, -3	Premature stop	
	-G--P--Q--F--P--S--R--G--G--*--P--*--V--L-	3 aa* + stop		
13 (wt)	GGACCTCAGTTCCCCT**+178bp**----------------------TCCTGG	+178, -23	Premature stop	Albino
	-G--P--Q--F--P--C-* -F--P--G	1 aa* + stop		
	**3’ CRISPR →**			
wt	TTATTGTGGGAACAAGAAATTCGAGAACTAACT*GGG*GATGAGAACTTC			
	-L--L--W--E--Q--E--I--R--E--L--T--G--D--E--N--F-			
1	TTATTGTGGGAACAAGAAATTCGAGAAC-----GGGGATGAGAACTTC	-5 bp	Premature stop	Black
	-L--L--W--E--Q--E--I--R--E--R--G--*--E--L--	2 aa* + stop		
10	TTATTGTGGGAACAAGAAATTCGAGAACTAA--GGGGATGAGAACTTC	-2 bp	Premature stop	Mosaic
	-L--L--W--E--Q--E--I--R--E--L--R--G--*--E--L--	2 aa* + stop		
11	TTATTGTGGGAACAAGAAATTCGAGAACTA---GGGGATGAGAACTTC	-3 bp	In frame deletion	Non-albino
	-L--L--W--E--Q--E--I--R--E--L- -G--D--E--N--F-	1 aa deletion		

The HMA profiles from tail DNA of these pups indicated indels in 6/13 (46%) mice at the 5’ CRISPR target region and 3/13 (23%) mice at the 3’ CRISPR target region. These results, similar to the results seen in cultured blastocysts, suggest that the 5’ CRISPR/sgRNA was more efficient in creating mutant alleles in comparison to the 3’ CRISPR/sgRNA.

We used tail genomic DNA from these F_0_ animals to look for the presence of indels using the heteroduplex mobility assay. Based on the HMA profiles, we could infer the presence of indels in 2/7 black mice, 2/3 mosaic mice, 1/1 non-albino mouse, and 2/2 albino mice (Table 1). Multiple clones from PCR amplicons showing HMA profiles were sequenced to identify the genetic lesions resulting from NHEJ. We obtained 8 mutant alleles corresponding to the 5’ CRISPR target region and 3 mutant alleles in the 3’ CRISPR target region (Table 2). Sequences obtained from the non-albino founder and one albino founder indicated bi-allelic conversion. The bi-allelic changes were unambiguously identified due to the distinct sequences of the Tyr locus (wt and c-2j) in the founder animals.

Reference sequences for the wildtype and c-2j alleles are shown in the top rows. Protospacer sequences are underlined and PAM sequences are highlighted in *italics*. The identity of the modified allele/chromosome is indicated by wt for wildtype allele and Tyr^*c-2j*^ for the albino allele in parentheses. Base insertions are in bold and deletions are indicated by dashes. Number of base insertions (+) and deletions (-) are shown in a separate column. Premature stop codons result in non-native amino acid incorporation, which are indicated by asterisk (*).

In order to facilitate easy phenotypic characterization of germline-transmitted mutations, we bred the mosaic, non-albino and albino F_0_ mice with *Tyr*^*c*-2j/c-2j^ albino mice. We were able to obtain F1 mice from the mosaic animals, the non-albino mouse and the 2 albino F_0_ mice (Table 1). We obtained F1 mice that carried all the alleles identified in the founder animals.

### Non-albino founder carries two related hypomorphic alleles–*dhoosara* and *chandana*

**Fig 2:**
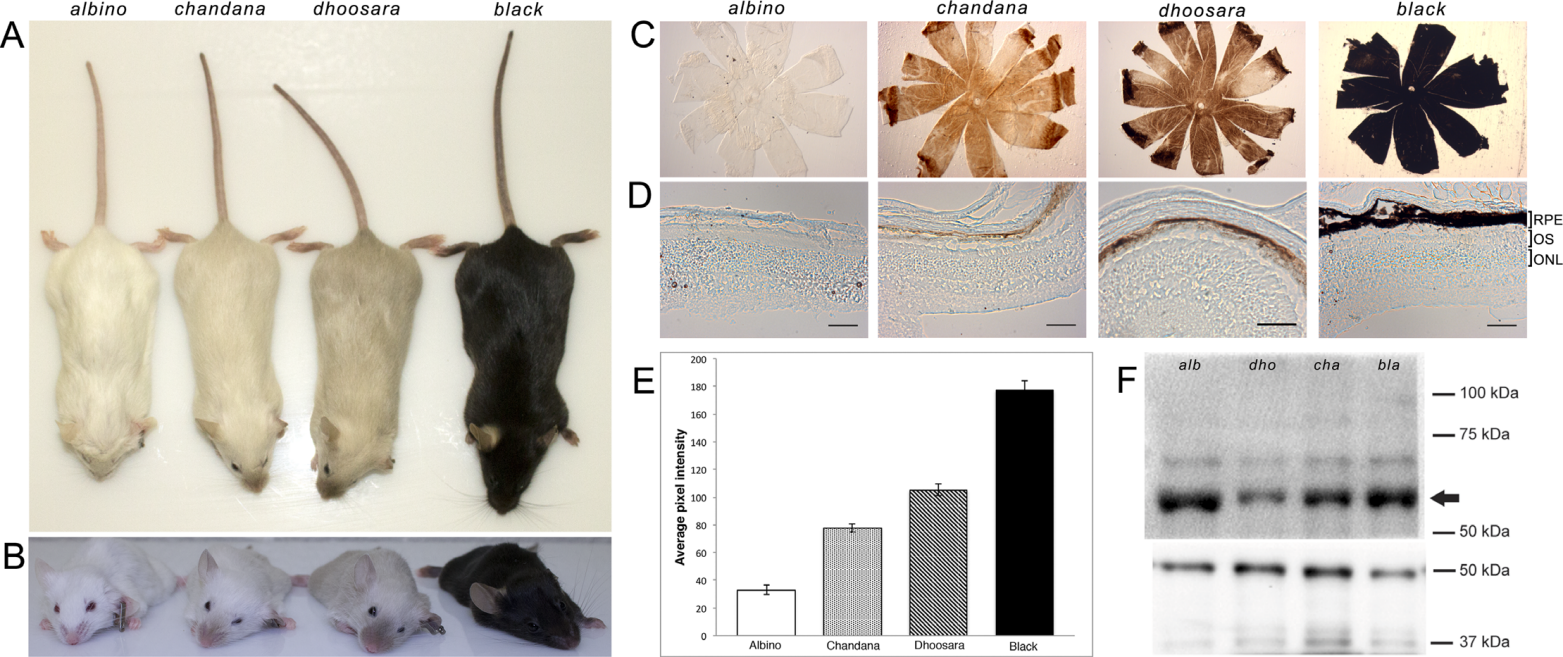
Comparison of *dhoosara* and *chandana* with the albino and black/wild type mice. (A) Dorsal view showing the coat color of albino, *chandana*, *dhoosara*, and black animals. Increasing levels of pigmentation can be seen in the hind limbs and tips of tails as well. (B) Frontal view of the face with increasing pigmentation in the nose region. Differences in the eye color can be noted. (C) Brightfield images of RPE wholemounts from Albino, *chandana*, *dhoosara* and Black mice. (D) Brightfield images of retinal cryosections from Albino, *chandana*, *dhoosara* and black mice. The RPE layer is the only layer within the eyeball that contains pigment. Scale bar = 50 μm. (E) Mean gray values (quantified in ImageJ) obtained from the RPE wholemounts reflecting relative intensity of RPE cell pigmentation. Results are presented in a bar chart with standard error of mean used for error bars. All groups are significantly different from each other (p<0.05, t-test). (F) Western Blot of tyrosinase protein isolated from skin of albino (alb), *chandana (cha)*, *dhoosara (dho)*, and black animals (arrow points to the 60 kDa tyrosinase band); 50 kDa tubulin protein (lower panel) is used as a control.

Of the different mutant alleles generated, a non-albino founder animal carried two related alleles with in-frame deletion mutations. Both alleles transmit through the germline and display dominance over the Tyr^*c-2j*^ allele. These alleles, when present with the *c-2j* allele, result in animals with distinct pigmentation (Fig 2A and 2B). Mice carrying only the 15 bp deletion allele (5’ CRIPSR target) had a noticeably darker pigmentation in comparison to the mice carrying both the 15 bp (5’ CRIPSR target) and 3 bp (3’ CRIPSR target) deletions. We name the darker pigmented phenotype as *dhoosara* (Sanskrit for gray) and the lighter pigmentation phenotype as *chandana* (Sanskrit for sandalwood). The body coat pigmentation in both *dhoosara* and *chandana* is lighter than that found at the extremities—nose/snout area, ears, limbs and the tail (Fig 2A and 2B).

### *dhoosara* and *chandana* have different pigmentation levels in RPE cells

Whole mount eye cups from each animal group (Fig 2C) indicated the differences in pigmentation both in the central Retinal Pigment Epithelium (RPE) as well the peripheral sclera and choroid. Comparison of the whole mounts from each group clearly shows the complete absence of any pigmentation in the Tyr^*c-2J*^ mutants and presence of black pigmentation in the wildtype, with the retinal sections prepared from *dhoosara* and *chandana* mice displaying observable differences in the pigmentation of the RPE cell layer (Fig 2D). Grayscale values measured from RPE whole mounts demonstrate significant differences in RPE pigmentation between all groups (p<0.05, t-test) (Fig 2E).

### Tyrosinase protein is expressed in both *dhoosara*a and *chandana*

To assess protein expression resulting from these alleles, we extracted and analyzed total protein from skin of adult mice by Western Blotting. Tyrosinase protein expression was detected in the skin of albino, *chandana*, *dhoosara*, and black animals (Fig 2F).

## Discussion

The ability to target specific genes and induce site-specific mutations in their sequences has been made possible with the emergence of designer nucleases over the last decade [13, 14]. The CRISPR-Cas9 system has accelerated this process in an unprecedented manner. Several reagents and resources have been generated and are now readily available to the entire research community ([15]; http://www.addgene.org/CRISPR/).

Using a simple workflow, we tested the efficiency of nuclease activity of CRISPR/sgRNAs and generated several mutant alleles in the mouse *Tyrosinase* (*Tyr*) gene. Targeting two distinct sites in exon 1 of the mouse *Tyr* gene with two validated CRISPR/sgRNAs resulted in mutations at both sites that were successfully transmitted through the germ line. We found several alleles with indels at the two individual CRISPR target sites, but we did not observe any large deletion events that would eliminate the intervening sequence between the two CRISPR target sites in the animals we analyzed.

One founder with a non-albino pigmentation phenotype carried two hypomorphic alleles in addition to a null allele, alluding to the fact that it is a genetic mosaic. The subtle differences in pigmentation made it hard to distinguish the phenotypes visually. The hypomorphic alleles are related, with one of them having an in-frame 15 bp deletion (*dhoosara*) and the other with an additional in-frame 3 bp deletion (*chandana)*. Since both the alleles have small in-frame deletions, we predicted that protein expression would not be affected. Western blot analysis using a rabbit polyclonal antibody against human tyrosinase (aa 20–340) showed that the protein is present in all cases. While the loss of function c-2j allele with an R77L substitution shows an albino phenotype, *dhoosara* with missing residues (resulting from the 15 bp sequence in close proximity to residue R77) shows reduced pigmentation. Since protein expression does not seem to be affected, the mutant protein is likely to have decreased enzymatic activity. This is further accentuated in the case of *chandana*—with an additional loss of Threonine at position 244 (due to a 3bp deletion) there is a further decrease in pigmentation even though the protein is expressed. Since 244T is in proximity to the Cu^2+^ binding catalytic site, its absence is likely to influence the catalytic site resulting in suboptimal enzyme function. The additive effect of these two in-frame deletions in *chandana* is clearly noticeable with pigmentation on the body and extremities. The presence of slightly darker pigmentation at the extremities, in comparison to the body, suggests that the mutant proteins are likely to be temperature sensitive, similar to that seen in the *siamese* mutation also found in the Himalayan mouse [16, 17]. Since the extremities are likely to be cooler than the body, stability of the temperature sensitive mutant proteins may be higher resulting in higher enzymatic activity. The differences in pigmentation seen in *dhoosara* and *chandana*, in comparison to albino and wildtype animals can also be seen in the eye cup, and especially in the retinal pigment epithelial cells.

**Fig 3:**
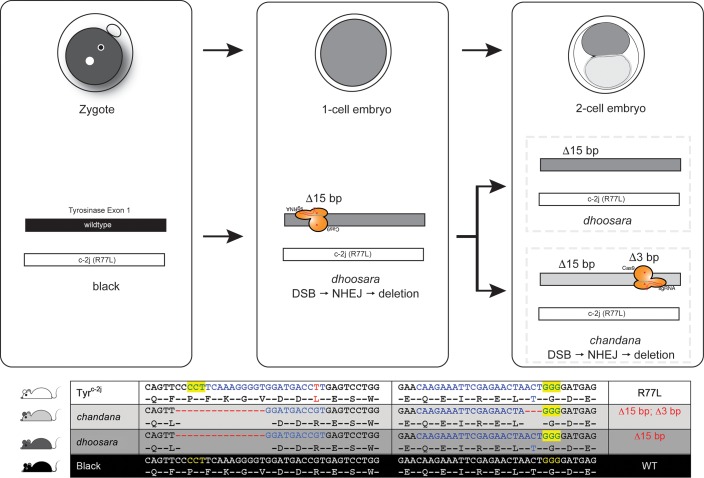
Schematic of possible sequence of events producing related *dhoosara* and *chandana* alleles. CRISPR-Cas9 nuclease activity in the zygote at the 5’ target site on the wildtype chromosome results in a 15 bp deletion. After the cell division, nuclease activity persists and creates a DSB at the 3’ target site on the previously modified chromosome. NHEJ repair in this daughter cell results in a second, 3 bp deletion.

Repair events at every double strand break site by the NHEJ mechanism are independent of each other. Therefore, it is unlikely to generate the exact same indel in two separate instances. Presence of the same indel in two alleles in a single founder animal can be explained by the persistence of CRISPR-Cas9 nuclease activity during subsequent embryonic cell divisions (Fig 3). In the first instance, a 15 bp deletion occurs after the action of the 5’ CRISPR and subsequent NHEJ repair. After cell division the 3’ CRISPR causes a second, 3 bp, deletion on the previously modified chromosome in only one of the daughter cells. As a result, the daughter cells have two distinct, but related, alleles. This points to the possibility that the CRISPR-Cas9 nuclease activity can be seen beyond the 1-cell/zygote stage, and exemplifies the mosaic nature of founder animals generated using these nucleases.

We describe two novel, but related alleles of the mouse *Tyrosinase* gene, *dhoosara* and *chandana* that cause hypomorphic non-albino pigmentation in mice. Identification of *dhoosara* and *chandana* has added new information to the list of hypomorphic alleles previously described (S1 Table). This study showcases the utility of CRISPR-Cas9 system in generating mutations in specific domains in mouse genes. As alluded by Yen and co-workers [2] visual phenotypes and genetic sequence information of the alleles throw light on the mosaic and allele complexity in potential founder animals. Targeting specific regions of the gene in conjunction with the possibility of producing several mutations due to NHEJ, this enhances the chances of identifying novel, domain-specific alleles.

## Supporting Information

S1 Table Hypomorphic alleles of the Tyr gene. Previously reported hypomorphic alleles of the mouse Tyr gene with the MGI IDs, allele names/synonyms, nucleotide change (nt), resulting amino acid change (aa), and reference (PMID). The albino 2 Jackson (c-2j) null allele is shown for comparison. Albino 2 Jackson (c-2j) allele is shown as a reference null mutation. Commonly used names, MGI IDs, nucleotide (nt) and amino acid (aa) changes for each allele is provided. References to available published accounts are indicated by PMIDs.
(DOCX)
