# Supplementary material for: Novel Hypomorphic Alleles of the Mouse Tyrosinase Gene Induced by CRISPR-Cas9 Nucleases Cause Non-Albino Pigmentation Phenotypes
Source: PLoS One. 2016 May 25;11(5):e0155812. doi: 10.1371/journal.pone.0155812 (PMC4880214; doi:10.1371/journal.pone.0155812)
Supplement: S1 Table — Previously reported hypomorphic alleles of the mouse Tyr gene with the MGI IDs, allele names/synonyms, nucleotide change (nt), resulting amino acid change (aa), and reference (PMID). The albino 2 Jackson (c-2j) null allele is shown for comparison. Albino 2 Jackson (c-2j) allele is shown as a reference null mutation. Commonly used names, MGI IDs, nucleotide (nt) and amino acid (aa) changes for each allele is provided. References to available published accounts are indicated by PMIDs. (DOCX) [file pone.0155812.s001.docx]

**Table S1. Hypomorphic alleles of the Tyr gene.**

| **Name** | **MGI ID** | **Synonyms** | **nt** | **aa** | **PMID** |
| --- | --- | --- | --- | --- | --- |
| albino 2 Jackson | 1855985 | c^2-j^ | G291T | R77L | 8921397 |
| chinchilla | 1855977 | c^ch^, c^r^ | G1444A | A482T | 2118105 |
| chinchilla-mottled | 1855980 | c^m^, c^22H^ | Rearrangement in regulatory region |  | 15572362 |
| extreme dilution mottled | 2683485 | c^em^ | C1220T | T373I | 15572362 |
| himalayan | 1855979 | c-h | A1259G | H420R | 2567165 |
| extreme dilution | 1855978 | c^d^, c^e^ |  |  |  |
| acromelanic | 1855983 | c^a^ |  |  |  |
| platinum | 1855981 | c^P^ | A1519T | K507* | 8654502 |
| albino 44 Harwell | 1856307 | c^44H^, dark-eyed albino | G519T | S128I | 8197131 |
